# Interventions for health workforce retention in rural and remote areas: a systematic review

**DOI:** 10.1186/s12960-021-00643-7

**Published:** 2021-08-26

**Authors:** Deborah Russell, Supriya Mathew, Michelle Fitts, Zania Liddle, Lorna Murakami-Gold, Narelle Campbell, Mark Ramjan, Yuejen Zhao, Sonia Hines, John S. Humphreys, John Wakerman

**Affiliations:** 1 Menzies School of Health Research, Charles Darwin University, Alice Springs, Australia; 2 Poche SA & NT, Flinders Northern Territory, Flinders University, Alice Springs, Australia; 3 Flinders Northern Territory, Flinders University, Darwin, Australia; 4 Northern Territory Department of Health, Darwin, Australia; 5 Flinders Northern Territory, Flinders University, Alice Springs, Australia; 6 The Centre for Remote Health: A Joanna Briggs Institute Affiliated Group, Alice Springs, Australia; 7 Monash Rural Health, Monash University, Bendigo, Australia

**Keywords:** Health workforce, Incentives, Remote, Retention, Rural, Turnover, Underserved, Personnel

## Abstract

**Background:**

Attracting and retaining sufficient health workers to provide adequate services for residents of rural and remote areas has global significance. High income countries (HICs) face challenges in staffing rural areas, which are often perceived by health workers as less attractive workplaces. The objective of this review was to examine the quantifiable associations between interventions to retain health workers in rural and remote areas of HICs, and workforce retention.

**Methods:**

The review considers studies of rural or remote health workers in HICs where participants have experienced interventions, support measures or incentive programs intended to increase retention. Experimental, quasi-experimental and observational study designs including cohort, case–control, cross-sectional and case series studies published since 2010 were eligible for inclusion. The Joanna Briggs Institute methodology for reviews of risk and aetiology was used. Databases searched included MEDLINE (OVID), CINAHL (EBSCO), Embase, Web of Science and Informit.

**Results:**

Of 2649 identified articles, 34 were included, with a total of 58,188 participants. All study designs were observational, limiting certainty of findings. Evidence relating to the retention of non-medical health professionals was scant. There is growing evidence that preferential selection of students who grew up in a rural area is associated with increased rural retention. Undertaking substantial lengths of rural training during basic university training or during post-graduate training were each associated with higher rural retention, as was supporting existing rural health professionals to extend their skills or upgrade their qualifications. Regulatory interventions requiring return-of-service (ROS) in a rural area in exchange for visa waivers, access to professional licenses or provider numbers were associated with comparatively low rural retention, especially once the ROS period was complete. Rural retention was higher if ROS was in exchange for loan repayments.

**Conclusion:**

Educational interventions such as preferential selection of rural students and distributed training in rural areas are associated with increased rural retention of health professionals. Strongly coercive interventions are associated with comparatively lower rural retention than interventions that involve less coercion. Policy makers seeking rural retention in the medium and longer term would be prudent to strengthen rural training pathways and limit the use of strongly coercive interventions.

**Supplementary Information:**

The online version contains supplementary material available at 10.1186/s12960-021-00643-7.

## Background

Retaining healthcare workers in rural and remote areas is a global problem [1]. Rural and remote health worker retention is crucial for continuity of care and the development of strong professional relationships between health providers and patients which are vital for improving health outcomes of vulnerable populations [2, 3].

Rural and remote populations, however delineated, even in high-income countries (HICs) such as Australia, USA and Canada [4–6], have a range of health vulnerabilities and frequently experience substantial disparities in health outcomes due to socio-economic factors, increased health risk factors and poorer access to health care compared to metropolitan populations [4, 7]. A high proportion of Indigenous peoples live in remote and rural areas, and they experience considerably poorer health outcomes than non-Indigenous citizens [8]. Recent health care system performance rankings for Australia, Canada and USA reveal poor access (4th, 10th and 11th respectively out of 11 countries) and equity rankings (7th, 9th and 11th respectively) [9]. Improved retention of health professionals in non-metropolitan areas would have lasting positive impacts on the health and wellbeing of rural and Indigenous populations.

Prior reviews suggested that health professional education delivered in rural areas is positively associated with rural retention, although participating in rural training may reflect pre-existing intention and motivation for rural practice rather than the intervention itself increasing rural retention [10, 11]. Many of the positive and negative intrinsic and extrinsic motivators are either personal or professional support factors which may be modifiable [12]. Despite this, and the World Health Organization (WHO) recommending a number of personal and professional support interventions to increase retention, there is a lack of evidence of their effectiveness and cost-effectiveness [13]. While coercive regulatory interventions, including financial incentives with return-of-service (ROS) requirements, are effective short-term recruitment strategies, there is little evidence of their long-term positive impact on rural or remote health workforce retention [14–16]. Financial retention incentives for individuals without ROS requirements are prevalent. WHO recommends offering increased allowances, grants for housing, increased paid recreational leave, and assistance with transport [13]. However, the evidence from HICs about the effectiveness of financial incentives (with no ROS obligation) is lacking.

Given these significant gaps in our understanding, this review aims to update existing evidence [17] by examining associations between interventions designed to retain health workers in rural and remote areas of HICs and quantifiable workforce retention outcomes.

## Methods

To ensure that no other research group had already undertaken the work, scoping of existing retention reviews included a preliminary search of PROSPERO, MEDLINE, the Cochrane Database of Systematic Reviews and JBI Evidence Synthesis. Four review papers were found that either needed updating or had a much narrower scope than this review [1, 17–19].

This systematic review accords with the Joanna Briggs Institute (JBI) methods for systematic reviews of aetiology and risk evidence [20] and followed an a priori published protocol which more fully describes methods and definitions [21]. However, studies reporting job satisfaction without direct turnover or retention outcomes were excluded [22], and we did not use the Grading of Recommendations, Assessment, Development and Evaluation (GRADE) approach for grading the certainty of evidence.

### Inclusion criteria

Inclusion and exclusion criteria are summarised in Table 1. Studies were confined to 2010 or later because of available substantive reviews that synthesised the evidence relating to retention up to that time [1, 11].

**Table 1:** Inclusion and exclusion criteria

	Include	Exclude
Year published	2010 or later	Prior to 2010
Participant types	Medical doctors, nurses, midwives, pharmacists, allied health professions, or health professionals generally	Other types of workers in the health sector or in other sectors
Countries	High-income country as per World Bank criteria [23], or if mixed income countries then data for high-income countries reported separately	Not high income or not reported separately
Exposure	Interventions, support measures or incentive programs implemented (or simulated) with the intention of increasing retention or reducing turnover	No intervention (or simulation) designed to impact on retention or turnover
Setting	Rural or remote as defined by ASGS (Australia) or equivalent national classification system or study's own description of being rural or remote	Not rural or remote
Outcomes	PRIMARY: Mean or median length of employment; survival probabilities; hazard, odds or relative risk ratios for staying/leaving rural; stability rates; settlement rates. (profile = retention in rural/remote area or community) SECONDARY: Vacancy rates; unfilled positions; turnover numbers or rates; attrition or wastage rates; rate of leaving before end of contract; intention to stay/leave; intention to return	Lack of quantifiable primary or secondary outcomes, job satisfaction
Study types	Analytical observational studies (prospective and retrospective cohort studies, case–control studies, cross-sectional studies); descriptive observational studies (case series, descriptive cross-sectional studies)	Qualitative studies
Language	English	Non-English

### Search strategy

A three-step search strategy was used to locate both published and unpublished studies [21]. The searches were undertaken 11–12 April 2019 and repeated on 1 July 2020 to capture any additional published studies. MEDLINE (OVID), CINAHL (EBSCO), Embase, Web of Science, Scopus, and Informit databases were searched as were ProQuest Dissertations and Theses, Trove and MedNar and the websites of government and peak non-government organizations. The MEDLINE search strategy is available as an Additional file 1.

### Study selection

**Fig. 1:**
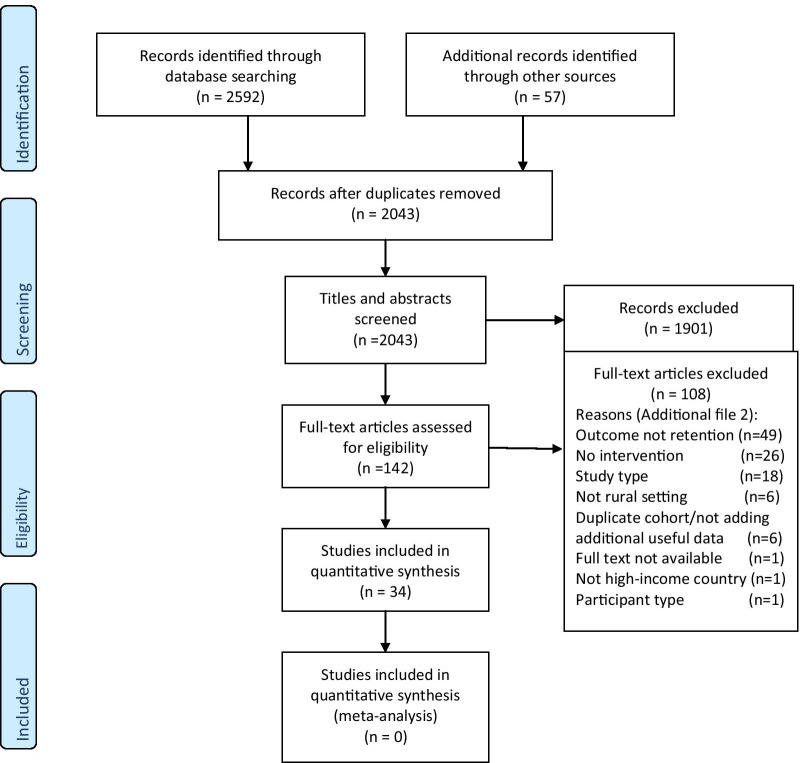
PRISMA flow diagram of search and study selection process [24]

All identified citations were collated and uploaded into EndNote Version X9 (Clarivate Analytics, PA, USA) and duplicates removed. Titles and abstracts were screened by two independent reviewers against the inclusion criteria. Potentially relevant studies were retrieved in full and their citation details were imported into the Joanna Briggs Institute’s System for the Unified Management, Assessment and Review of Information (JBI SUMARI; JBI Adelaide, Australia). Using the inclusion criteria, the full text of each citation was assessed independently by two independent reviewers. In the few instances where more than one paper was from the same research study only one paper was included. Reasons for exclusion of full text studies were recorded and are reported in the Preferred Reporting Items for Systematic Reviews and Meta-analyses (PRISMA) flow diagram (Fig. 1) [24].

### Assessment of methodological quality

Eligible studies were critically appraised and scored by two independent reviewers for methodological quality using the appropriate JBI critical appraisal instrument [25]. Disagreements were resolved through discussion, or with a third reviewer.

### Data extraction

Data were extracted by two independent reviewers using the standardized data extraction tool from JBI-SUMARI. Study citation details, study objective, participant information, details of the setting/context, details of the retention intervention, and study results for the relevant outcomes were extracted.

### Data synthesis

The structure of the narrative synthesis of extracted data was based on categories for rural health workforce interventions used in the WHO Global Policy Recommendations [26]: education; regulatory interventions; financial incentives; and personal and professional support. A further category—health systems—was added, as proposed by Putri et al. [27].

## Results

### Study selection

The search strategy identified 2592 papers, with a further 57 papers identified from other sources (Fig. 1). After duplicates were removed 2043 papers remained. 1901 papers were excluded by title and abstract screening and 142 articles underwent full text assessment. 108 were excluded, leaving 34 articles. The main reasons for exclusion on full text review were a lack of quantifiable retention outcome measures, no intervention or ineligible study type (Additional file 2).

### Methodological quality

Most were cohort studies (29/34, 85%). Methodological quality of included studies was generally low (Additional file 3). The median score for included cohort studies was 16 out of a maximum of 22 (interquartile range 13–20). Only one-third of included studies applied appropriate statistical analysis, with less than half adjusting for key potential confounders. Many studies had no comparator group.

### Study characteristics

Study characteristics, participants and sample size, interventions, outcome measures and main findings are shown in Table 2. Of the 34 included studies, 13 (38%) were from Australia, 11 (32%) from USA, five from Canada and five originated from Nordic and nearby northern European countries. Most (*n* = 28, 82%) studies exclusively examined retention of doctors. Three studies were exclusively of nurses [28–30], one exclusively of dentists [31] and two studies included mixed health professions [32, 33]. There were a total of 58,188 participants or participant observations in included studies. Four studies were outliers in terms of their comparatively large sample size [34–37]. Most (*n* = 29, 85%) studies measured actual retention (or turnover), with the remainder measuring health professional preferences, intentions or simulating interventions. Actual turnover and retention were measured/defined over very variable periods of time: for example, one study measured only 6 months rural practice as being ‘long-term’ [38] whereas another documented up to 38 years of rural retention [39]. The outcome measures in 12 studies were retention rates [28–30, 33, 40–47], while five studies used survival probabilities or hazard ratios [37, 48–52], and a further five used odds or relative risk ratios for staying or leaving [31, 35, 36, 53, 54]. The turnover or retention profile used most frequently in included studies was at the level of rural or remote practice anywhere within a country [31, 35, 36, 38, 46, 47, 54–59]. Next most frequent was at the level of a largely rural jurisdiction [29, 41, 48, 50, 52] and rural practice within a jurisdiction [34, 40, 42, 49, 51], followed by turnover or retention within a rural community [33, 37, 45, 53].

**Table 2:** Characteristics of included studies

Study	Country	Participants and sample size	Study design	Intervention(s)	Outcomes measured	Main description of results
Carson et al. [32]	Iceland, Ireland, Norway, Scotland, Sweden, Greenland	1046 rural health professionals (doctors, nurses, allied health professionals)	Cross-sectional	Selection of rural background or rurally-schooled students; rural health professional training (rural pipeline)	Intention to stay with same rural organisation for at least the next 2 years (or until retirement if intending to retire within 2 years)	There was a significant relationship (*χ*^2^ = 3.98, *p* < 0.05) between having a rural background and intending to stay with the same rurally located organisation. Spending the majority of school education in a rural area was also significantly associated with intention to stay (*χ*^2^ = 8.7, *p* < 0.01). Health professionals working in outer rural areas who had undertaken some of their health professional training in a rural area were more likely to intend to stay than those who had no rural training (*χ*^2^ = 4.22, *p* < 0.05)
Chauhan et al. [55]	Canada	642 rural physicians	Cross sectional	Any length of rural training during medical school	Intention to leave rural practice within the next 2 years	There was no significant association between having had at least one rural experience of any length during medical school (versus no rural rotations at all) and intention to leave rural practice within 2 years
Cogbill and Bintz [39]	USA	19 rural General Surgeons who worked for Gundersen Health System since it commenced in 1978	Case report	A rural general surgery network designed as a sustainable model for delivery of general surgery services within a large rural region of the midwestern USA (Minnesota, Wisconsin, Iowa)	Mean number of months that currently employed General Surgeons have been practising in Gundersen Health System network	The study showed that of the 19 rural General Surgeons ever employed in the network 21% (*n* = 4) have retired, 53% (*n* = 9) continue to practice in the network, and only 26% (*n* = 5) left the network before retirement. The nine currently employed General Surgeons have been practising in the network for a mean of 88.0 months (SD 83.6; range 24 to 288 months). Six have practiced in one location for more than 20 years
Fleming and Mathews [48]	Canada	391 physicians who were initially licensed to practice in Newfoundland-Labrador (NL) between 1993–2004	Cohort	Trained locally at Memorial University (MMG) versus trained elsewhere in Canada (CMG) versus provisionally licensed international medical graduates (IMGs (Prov)) versus fully licensed international medical graduates (IMGs (Full))	Hazard of remaining in NL	Overall, CMGs, IMGs (Prov) and IMGs (Full) were 1.99 (95% confidence interval [CI] 1.28–3.10), 2.11 (95% CI 1.50–3.00) and 1.86 (95% CI 1.19–2.90) times more likely to leave NL, respectively, than locally trained MMGs. Physician group was the only significant covariate in the Cox regression analysis
Gardiner et al. [40]	Australia	361 rural General Practitioners (GPs) in South Australia	Cohort	Group and individual coaching by qualified psychologists and 6 weeks of email coaching over a 3-year period	Retention rate in rural general practice at two time points, 3 years apart	Despite having a much higher intention to leave rural general practice before coaching, only 6% left after coaching. In other words, 94% of participants stayed compared with 80% of the general rural GP population [*χ*^2^ = 4.89, *p* = 0.027]
Gaski and Abelsen [41]	Norway	388 Graduating medical students	Cohort	Medical internships early sign up versus raffle in the study area versus a comparison area with similar workforce issues	% former interns in the area staying in (rural) county as at April 2014 (Finnmark versus another rural county)	The proportion of interns who signed up early who still worked as physicians in the study area (29%) was twice as high as among the regular interns (15%) and interns in the comparison area (14%). None of the 59 physicians who had been early signup interns worked in any of the 15 remote municipalities in the study area
Gorsche and Woloschuk [42]	Canada	29 rural doctors working in an established rural practice in Alberta with written support from their regional medical director, who enrolled in the skills enrichment program March 2001–March 2005 and their 29 matched controls	Cohort	Skills enrichment program comprising training for acquisition of or maintenance of existing skills of at least 2 weeks’ duration but less than 1 year, with preceptor funding and locum support available and funded up to $80,000 per annum (pro rata)	Rural practice retention 5 years after the program	After 5 years, all 29/29 (100%) physicians in the enrichment group remained in rural practice compared with 22/29 (71%) of physicians who did not participate in the enrichment program: (Relative Risk 1.31; 95% CI 1.06–1.62). Two enrichment participants who left the province and their controls were excluded from analyses
Jamar et al. [56]	Australia	74 University of Adelaide Rural Clinical School domestic medical students 2003–2010	Cohort	Rural Clinical School education exposure comprising completing the whole of fifth (penultimate) year of clinical studies at a rural location	% of graduates who spent no time, 3 to 18 m and 2+ years in a rural area since graduation	Of the 72 survey responses analysed, 44% (*n* = 32) spent no time in a rural location, 28% (*n* = 20) spent 3 to 18 months in a rural area and another 28% (*n* = 20) spent two or more years in a rural area since graduation
Jamieson et al. [53]	Canada	480 University of British Columbia Family Medicine residency graduation cohorts from 1990 to 2007	Cohort	Rurally distributed postgraduate training sites versus training in metropolitan teaching centers	Retention in current community for more than 1 year	Amongst those who had been in their current community for more than 1 year, those who had postgraduate training in smaller rurally distributed sites were 36 times more likely to be working in a rural or regional practice than those trained in metropolitan sites (95% CI 12.2–108.5)
Johnson et al. [31]	Australia	397 dental graduates of University of Sydney 2009–2013	Cohort	Rural Clinical Placement Program comprising an opt in 1-month clinical placement in rural NSW in final year of dental school; previous rural life experience	Working in a rural location in both 2015 and 2017 versus not working in a rural location in both 2015 and 2017	Having had some form of rural experience prior to the rural clinical placement (PR 3.75 95% CI 2.75–5.11) and pre‐placement rural intentions (PR 3.54 95% CI 2.25–5.57) were significant independent predictors of an increased likelihood of working in a rural location in both 2015 and 2017
Kahn et al. [49]	USA	47 IMG physicians participating in Conrad program 1995–2003 who were assigned to a rural community for return-of-service	Cohort	Conrad program which allows thirty J-1 visa waivers each year in Washington state with participants having a ROS obligation of 3 years for primary care physicians and 5 years for specialists	Retention rate in rural areas after completing rural J-1 visa waiver ROS obligation	Of the 47 physicians who undertook their periods of service in a rural area, only 12 (26%) stayed in a rural area following completion of the ROS obligation
Kwan et al. [38]	Australia	729 medical graduates of University of Queensland (2002–2011)	Cohort	Rural clinical exposure for one (RCS-1) or two (RCS-2) years; bonded scholarship; rural background vs metropolitan background	Long term rural practice (LTRP) defined as ≥ 50% of the graduates’ primary place of practice since graduation being rural	Independent predictors of LTRP were Rural Background (OR 2.10 [95% CI 1.37 ± 3.20]), RCS-1 (OR 2.85 [95% CI 1.77 ± 4.58]), RCS-2 (OR 5.38 [95% CI 3.15 ± 9.20]) and having a bonded scholarship (OR 2.11 [95% CI 1.19 ± .76])
Li et al. [57]	Australia	1117 rural GPs who participated in the Medicine in Australia: Balancing Employment and Life (MABEL) survey in 2009	Cross-sectional and discrete choice experiment	Locum relief guarantee, retention payments, rural skills loading, family isolation/secondary school costs or retention grants for existing rural doctors	Probability of attracting rural General Practitioners to (hypothetically) choose to stay in rural practice	Increasing the level of locum relief guarantee, GP retention payments and rural skills loading from zero to the middle and high levels was associated with increased rural GP retention. In order from highest to lowest effect on retention were: guaranteed locum coverage for 6 weeks every 12 months (*β* = 1.51); 50% increase in retention payments (*β* = 1.36); guaranteed locum coverage for 4 weeks every 12 months (*β* = 0.85); rural skills loading payment increase by 20% (*β* = 0.82); 25% increase in retention payments (*β* = 0.54); and a rural skills loading payment increase by 10% (*β* = 0.33). One-quarter of rural GPs were not influenced by the rural incentive packages
Mathews et al. [50]	Canada	60 physicians who were trained at Memorial University Newfoundland and held ROS (Return-of-Service) agreements (1997 to 2009) and started practice in Newfoundland and Labrador (NL) 2000–2005 compared with all 67 other NL physicians who started practice in NL between 2000 and 2005	Cohort	Opt-in ROS agreements offered to medical students and junior doctors which included two types of bursaries: Family Medicine Bursary and Special Funded Residency Position (offering postgraduate training positions which are usually accepted by physicians who were unable to secure a position through the usual application process)	Retention of physicians in the Canadian province of NL at the end of the follow-up period (December 31, 2010)	Whether or not Memorial University Newfoundland-trained (MUN-trained) physicians received a ROS bursary was a significant predictor of leaving NL. ROS physicians were 3.2 (95% CI 1.4–7.1) times less likely to leave NL than non-ROS physicians. Amongst the 60 ROS and 67 non-ROS MUN-trained physicians, 10 (16.9%) ROS versus 28 (41.8%) non-ROS physicians left NL province by 2010 (*p* = 0.004)
McGrail and Humphreys [36]	Australia	3782 responses from GPs who responded to Medicine in Australia: Balancing Employment and Life (MABEL) survey at least twice between 2008 and 2012 (inclusive)	Cohort	Training location (Australian trained vs non- Australian trained); restrictions on access to provider numbers related to geographic location for International Medical Graduates (IMGs)	Annual location retention rates in regional, rural and remote areas and odds ratios for leaving a rural area	There was no significant difference in the risk of leaving rural practice for IMGs compared to Australian non-restricted graduates. This was true for IMGs whether or not they had restrictions on access to provider numbers limiting where they could practice
McGrail et al. [58]	Australia	610 Medicine in Australia: Balancing Employment and Life (MABEL) survey respondents (2008–2014 inclusive) who had completed GP training and were transitioning to independent practice	Cohort	Vocational training location (rural or metro); rural/metro origin; rural bonding (being contracted to work for part of their early career in a rural location)	Proportions of GPs working in rural and metropolitan locations during each of the first 4 years following completion of GP vocational training; proportions of rurally-trained GPs working in the same or a different rural community from that in which they completed their vocational training	The rural training pathway, regardless of childhood location, was extremely strongly associated with subsequent rural practice (ORs ranged from 29 to 92 in the first 4 years following completing GP training). The odds of rural practice for the rural training cohorts of GPs decreased with time. Rural bonding (OR 3.5–5.1) and rural origin (ORs 2.0–4.1) were also positively and significantly associated with rural practice in each of the first 4 years following completing GP training
Murray et al. [28]	USA	60 nursing staff employed by Bassett Medical Center in rural upstate New York, USA	Cohort	A Partnership for Nursing Opportunities opt-in Program which involved Bassett Medical Center collaborating with two different local colleges to better design postgraduate degree pathways that were attractive to nurse employees. The intervention included: fully paid tuition with return-of-service; flexible scheduling arrangements supporting full and part-time paid work; local teaching; academic advisor	Vacancy rates and annual turnover rates of nurses from the rurally located Bassett Medical Center	Licensed Practice Nurses turnover decreased from 16.8% in 2005 to 6.8% in 2009. There was no trend in reduced annual turnover evident for Registered Nurses, but Registered Nurse vacancy rates fell from 16.5% in 2005 to 4.3% in 2009
Nilsen et al. [29]	Norway	159 nursing students doing a bachelor’s degree course in Finnmark University and who graduated in 2002, 2004 and 2005	Cohort	Bachelor of Nursing program which offered off-campus training in rural areas near the students’ place of residence and using more flexible and team-based learning methods as an alternative to the usual on-campus training at a regional centre	Retention rate in Finnmark county (living and working) for at least 4 years after completing the study program	Off-campus training was associated with a considerably higher retention rate *n* = 37/40 (92.5%) in Finnmark county for graduates compared to on-campus training *n* = 83/119 (70%). The majority of the nurses who trained off-campus worked in rural or remote communities where nurse shortages were more pronounced
Norbye and Skaalvik [30]	Norway	233 Registered Nurses training at the Arctic University of Norway by a decentralised nursing education model	Cohort	Decentralized nursing education allowing part-time postgraduate study at proximate study centres to accommodate students’ family and work responsibilities	Nurse retention rates in rural areas after graduation from postgraduate studies	The majority [*n* = 190/233 (81.6%)] of nurses completing postgraduate education using the decentralised nursing education model continued to work in rural areas
Opoku et al. [51]	USA	240 physicians with an initial rural Nebraska county practice location and enrolled in J-1 visa or Nebraska Loan Repayment Program 1996–2012 inclusive	Cohort	J-1 visa program (waiver of the requirement for IMGs on J-1 visas to return to their home country in exchange for service in a health professional shortage area) and state loan repayment programs (loaning physicians up to $40,000 per annum for up to 3 years in exchange for service)	Hazard of leaving rural Nebraska (hazard ratio), average length of stay (years)	The average length of stay in rural Nebraska for J-1 visa waiver and state loan repayment physicians were 4.1 and 8.1 years, respectively (SD = 0.27 and 0.47, respectively) and for physicians who had completed the minimum obligatory period were 5.6 and 9.7 years (SD = 0.27 and 0.43, respectively) The likelihood of departure from rural Nebraska was higher for beneficiaries of the J-1 visa waiver program (HR = 3.76, 95% CI 2.02–6.98) compared to those receiving state loans
Patterson et al. [59]	USA	Family Medicine “1–2” Rural Training Track (RTT) residency programs that responded to survey providing information about 253 enrolled residents who graduated 2008–2014 inclusive	Cohort	“1–2” RTT Family Medicine residency program which includes up to 1 year of urban training and 2 years of rural training	% of RTT graduates who were practising in a rural location at 1 year, 2 years, and 3 years after finishing residency	Just under one-third (32.8%) of RTT graduates practiced in rural areas in their 1st year post graduation. The percentage practising in a rural location increased thereafter such that the percentage was above 35% in most of the 7 years post-graduation
Pepper et al. [61]	USA	693 physicians practising in the State of Wyoming and responding to a survey	Cross-sectional	Variation in malpractice insurance costs between different USA states	Intention to change practice in Wyoming in next 10 years: relocate within Wyoming, move out of Wyoming, stop patient care	The high cost of malpractice insurance in Wyoming was associated with planning to move out of state instead of moving within the state (OR 22, 95% CI 1.7–287.9). Of those planning to leave Wyoming, just under one half (44%) gave malpractice insurance costs as a reason
Playford et al. [60]	Australia	915 University of Western Australia graduates 2004–2010	Cohort	Training medical students at a Rural Clinical School; selecting 25% of students into medicine based on having a rural background; recipients of a Bonded Medical Place at medical school (a place in medical school in exchange for return of service in a district of workforce shortage) or a Medical Rural Bonded Scholarship (recipients of a bursary during medical school in exchange for return of service in a rural area following graduation)	Mean cumulative duration (in years) in rural practice, ratio of means	Rural origin Rural Clinical School participants had a cumulative duration of rural practice over 5 times higher than the urban origin/urban training reference group (Ratio of means 5.4, 95% CI 4.3–6.8). Urban origin and Rural Clinical School (Ratio of means 2.2, 95% CI 1.8–2.7) and rural origin and no Rural Clinical School (Ratio of means 2.9, 95% CI 2.2–3.8). Bonded graduates had longer mean cumulative duration of rural practice than non-bonded graduates (*p* < 0.0001)
Rabinowitz et al. [43]	USA	92 Jefferson Medical College graduates 1978–1986 inclusive who initially practised Family Medicine in a rural area	Cohort	Physician Shortage Area Program (PSAP) of Jefferson Medical College which is medical school rural program with features including: selection based on rural background, commitment to rural practice, faculty mentoring and career guidance, 6 week clinical placement in a small town in 3rd year, encouragement to take up rural preceptorship in 4th year and expectation of completing family medicine residency training	Numbers of PSAP and non-PSAP graduates who originally entered rural family medicine and were still practicing family medicine in the same rural area (including in five adjacent counties) in 2011	Of the 37 PSAP graduates who originally entered rural family medicine, 26 (70.3%) were still practicing family medicine in the same rural area in 2011. A significantly smaller proportion of the 52 non-PSAP graduates (*n* = 24, 46.2%) were practising in the same rural area (*p* = 0.02)
Renner et al. [33]	USA	66 health professionals who were participants in one of three Colorado loan repayment programs 1992–2007 and had fulfilled terms of service	Cohort	The Colorado loan repayment programs had varying rates of repayment of recipients’ educational debts (maximum of $35,000 per annum), duration of return of service commitments (0, 1 and 2 years) and eligibility based on health profession. Analysis was for participants in any of the 3 programs, with no comparison of outcomes of the different programs	Retention rate in a rural community, retention rate in the same rural community after completing their return of service commitment, percentage retained in the same rural community for 0–1, 2–4 and 5 or more years	Of the loan repayment recipients who had completed their service commitment at the time of the survey, 27 (64%) of the rural participants were still practising in a rural community compared to 23 (96%) of the urban participants who were still practicing in an urban community. Rural retention rates were not associated with past attendance at rural high schools or by intention to practice in a rural community regardless of loan repayment. Of the 36 loan repayment recipients who were still at their original site after completing their terms of service or having their loan paid off, 21 were rural participants. Of these who stayed, 10 (47.6%) had stayed 0–1 additional years, 8 (38.1%) had stayed 2–4 years, and 3 (14.3%) had stayed for 5 years or longer
Robinson and Slaney [44]	Australia	57 GP registrars trained in the rurally located Bogong Regional Training Network 2004–2009, Victoria	Cohort	Decentralised model of GP training in rural Victoria	Retention rate in rural general practice following training in Bogong region; retention rate in Bogong region following training in Bogong region	More than 42% of the GPs who had completed their GP vocational training remained in rural general practice and 32% remained in the Bogong region
Rodney et al. [45]	USA	80 Obstetrics and Gynaecology Family Medicine fellows 1992–2010 inclusive	Cohort	1-year post-residency obstetrics fellowship undertaken in a rural location	Service in a rural community for at least 2 years	Rural service of at least 2 years occurred among 47/74 (64%) of the graduates
Ross [46]	USA	62 graduates of a rural Family Medicine residency program in Oregon (Cascades East Family Medicine Residency Program) 1994–2009 inclusive	Cohort	Rural residency in Oregon undertaken in a community of population size 42,000	Length of stay in first practice location, length of stay in current (2009) practice location	Graduates spent a mean 3.5 years at their first practice location and 3.7 years at their current practice location. Only 40% (*n* = 25/62) of graduates had relocated their practices at least once since graduation. Half of all program graduates remained currently working in rural communities (defined as having a population size of less than 25,000 and located more than 25 miles from major centres)
Russell et al. [37]	Australia	2783 rural GPs working in New South Wales 2003–2012, (excludes locums, GPs in border towns or offshore and GP Registrars)	Cohort	Holding rights to admit patients to the local hospital as a Visiting Medical Officer, having restrictions on access to a provider number (conditional registration)	Hazard of leaving a rural community for more than 3 months	Rural NSW GPs with conditional registration were more likely to leave (HR 1.49, 95% CI 1.24–1.79) a rural community than those with no such restriction. Having no public hospital admitting rights (HR 1.49, 95% CI 1.30–1.71) was associated with a higher risk of leaving rural NSW communities
Straume et al. [52]	Norway	76 postgraduate trainee physicians in Family Medicine or Public Health 1995–2008 inclusive	Cohort	A 5-year postgraduate training model offered to interns in either a Family Medicine or Public Health training group which included professional support provided through group tutorials for 2–3 years, in-service training opportunities provided in rural areas and completion of compulsory courses	Retention rates in Finnmark 5 years after completion of compulsory group tutorage component of the postgraduate training	Amongst the 15 Public Health doctors who had completed the group tutorage more than 5 years ago, 10 (66.7%) were still working in the county. Overall 28/40 (70%) of the Public Health graduates were still working in Finnmark in 2009. Amongst the 37 Family Medicine doctors who had completed the group tutorage more than 5 years ago, 24 (64.9%) were still working in the county. Overall 53/72 (73.6%) of the Public Health graduates were still working in Finnmark in 2009
Wearne et al. [47]	Australia	24 General Practice Registrars enrolled in the Remote Vocational Training Scheme (RVTS) 1999–2005 inclusive	Cohort	RVTS program which trains doctors in general practice in remote communities using distance education and supervision	Retention rates of RVTS graduates still working in rural areas	The majority [*n* = 17/21 (81%)] of RVTS graduates continue to work in rural areas (defined as Rural, Remote and Metropolitan Areas 3–7) 2–8 years after completing the program
Woolley et al. [54]	Australia	529 James Cook University (JCU) Queensland graduates 2005–2011 inclusive	Cohort	JCU Rural Clinical School (Darwin), rural generalist (residency) training, outer regional or remote location for internship	Prevalence odds ratios (POR) for practising in a remote location for 1 year or more from Postgraduate Years (PGY) 4–10	Forty-seven (8.9%) of the 529 JCU medical graduates in the first seven cohorts had practised for at least 1 year in a remote location between PGY 4 and 10. The likelihood of JCU medical graduates practising in a ‘remote’ location from PGY 4 to 10 was associated with undertaking rural generalist training (*p* < 0.001; POR = 17.0), attending the Darwin clinical school in years 5–6 (*p* = 0.005; POR = 4.7) and undertaking an internship based in an outer-regional or remote community (*p* = 0.006; POR = 3.5)
Yong et al. [35]	Australia	Australian GPs responding to the Medicine in Australia, Balancing Employment and Life (MABEL) survey 2008–2014 inclusive and providing data for 4,822 exits from rural locations that were eligible for receiving General Practice Rural Incentives Program (GPRIP) incentive payments	Cohort	Changes to the GPRIP policy made some locations newly eligible for rural retention incentives and increased retention incentives for other rural locations already eligible	GP exits (turnover) from always eligible locations versus newly eligible locations under GPRIP	For both newly eligible and always eligible rural locations, GPRIP appeared to have no statistically significant effects on GP relocation exits (turnover)
Zhou [34]	USA	Physicians licensed in North Carolina and working in rural areas. Sample size for the rural retention simulation study was unclear. The overall study sample was 29,908 unique physicians working in North Carolina contributing 165,688 person-year observations	Cohort and simulation	Loan forgiveness programs, increased physician reimbursement rate, change in the composition of health care providers: changes to mid-level practitioner supply, increase supply of RNs, Medicaid and Medicare expansion in rural areas	Retention in rural areas of North Carolina (excludes becoming inactive, moving out of North Carolina)	Simulated loan forgiveness programs (representing an almost doubling of income for rural physicians) was associated with a small (up to 3%) decrease in the probability of them moving away from the same rural area after 1 or 2 years of service, while a 5% increase in rural county physician salary decreased the probability of moving away from the same rural area after 2 years by 7%. There was a decreasing marginal return on the probability of moving away from the same rural area with salary increases of 10 and 20%. A 5% increase in mid-level practitioners in rural counties increased the probability of rural physicians moving away after 2 years by 8%, while a 5% increase in RNs in rural counties decreased the likelihood of physicians leaving rural areas by 3%. Medicaid and Medicare expansion in rural areas were associated with increased physician movement away from their rural county of service after 2 years by 10% and 11% respectively

### Findings

A meta-analysis could not be conducted because the studies were highly heterogeneous, reporting different interventions and retention outcomes. Hence, a narrative approach was taken.

### Education

Twenty-one studies investigated the impact of educational interventions, including: selecting university students with rural backgrounds [31, 32, 38, 58, 60]; location of university training and its duration (rural, within a jurisdiction, within the same country) [31, 32, 36, 38, 43, 48, 54, 56, 58, 60]; multi-faceted interventions providing support for advancing education to a degree level (paid tuition with ROS obligation, scheduling flexibility, locally accessible, academic support locally; off-campus, decentralised education opportunities) [28–30]; location of postgraduate training [44–47, 52, 53, 58, 59]; and multi-faceted interventions supporting skills training for fully qualified rural health professionals (backfill, payment for supervisors) [42]. One study measured retention intentions; the remainder measured actual retention or turnover outcomes [32].

Selecting university students from rural backgrounds was consistently associated with increased rural retention [31, 32, 38, 58, 60]. Undertaking 1 or more years of university health training in non-metropolitan regions was associated with longer retention [43, 48, 54, 56, 60]. The retention of students who chose longer duration of rural training exposure during their basic training (2 years) were approximately double (odds ratio 5.38, 95% CI 3.15–9.20) those of students who chose 1 year (odds ratio 2.85, 95% CI 1.77–4.58) [38]. Opting in to a much shorter 1-month rural clinical placement during final year as a dental student was associated with increased prevalence of working rurally as a dentist in both 2015 and 2017 (prevalence ratio 1.93, 95% CI 1.19–3.15) compared to those who had no rural clinical placement [31]. Undertaking basic health professional training at an international versus a domestic university had no association with rural retention of graduates in two studies [36, 48]. One study showed internationally trained doctors were more likely to leave the province than graduates trained within that province [48].

Three studies with 392 nurses [28–30] showed that supporting existing employees of rural health services and rural residents to undertake further university study using distributed models of education in conjunction with paid tuition, flexible schedules which allowed concurrent part-time or full-time paid employment, local teaching and tailored academic support was associated with high retention or low turnover and vacancy rates. Annual turnover of Licensed Practice Nurses at a US rural medical centre decreased from 16.8 to 6.8% following program implementation [28]. Nilsen reported 4-year nurse retention of 92.5% in a northern Norwegian county in an off-campus rural training group, compared to 70% for those who trained on-campus [29].

Nine studies investigated associations between retention and rural or remote location of postgraduate training. Two studies, specifically examining internship location and retention [41, 54], showed there was considerable variation in the proportion of postgraduates retained in rural or remote locations (ranging from approximately 0.35 up to 0.8). Undertaking postgraduate training in smaller rural sites (population < 10,000) was associated with marked increase in retention (odds ratio 36, 95% CI 12–109) [53]. A positive association between rural or remote postgraduate training and retention was reported to be stronger amongst rural origin registrars [58]. One Canadian study found that postgraduate (residency) training in a largely rural province was not a significant predictor of retention in the province, after adjusting for undergraduate training in that province [48]. An Australian study found an association between postgraduate rural generalist training (training which includes developing advanced skills, for example in Aboriginal health) and remote retention [54].

A small study of a skills enrichment program for fully qualified rural physicians, with provision for backfill and funding for preceptors for up to 1 year, reported all 29 were retained 5 years later, whereas significantly fewer matched physicians (22/29) not participating in the program were retained (risk ratio 1.3 95% CI 1.1–1.6) [42].

### Regulatory interventions

Nine studies examined the effectiveness of regulatory interventions on retention of doctors. One study simulated the impact of different types of health workers on doctor turnover. Increasing access to mid-level practitioners such as Physician Assistants and Advanced Nurse Practitioners was associated with a significantly increased probability of physicians moving away from the area after 1 or 2 years of service as these providers are a substitute for physicians [34]. In contrast, increasing rural supply of registered nurses, who provide services that complement those of physicians, was associated with a significant decrease in the likelihood of rural physicians leaving [34].

Eight studies investigated interventions which required service in rural areas (for a varying length of time) in return for a benefit. Several studies demonstrated that interventions comprising ROS in a rural area in exchange for highly valued visa waivers or access to professional licenses or provider numbers were associated with comparatively low rural retention/high turnover, especially once the ROS period was complete. Visa waiver recipients in Nebraska, USA, were almost four times more likely to leave rural areas of the state than state loan repayment recipients [51]. In contrast, the loan repayment recipients remained in rural areas for many years, with more than half still there 17 years later. Half of the visa waiver recipients left within 2 years of completing the 3-year minimum obligatory period. A study of loan repayment recipients who had completed ROS obligations in Colorado, USA, found that approximately two-thirds of rural recipients were still practising in a rural community compared to almost 100% of urban recipients who were still practising in an urban community [33]. Almost half (*n* = 10/21) of the loan repayment recipients who had finished their rural service obligations stayed less than 1 year beyond their ROS obligation [33]. One study found that international medical graduates (IMGs) who had yet to complete their ROS obligations had a substantially higher hazard of turnover than IMGs without locational restrictions [37]. Another study, however, found no statistically significant difference in the risk of leaving rural for IMGs, whether they had work location restrictions or not, compared to non-restricted graduates [36].

The intervention groups in two studies were subject to different types of regulatory interventions [50, 60]. Playford et al. were unable to differentiate the association of retention with two different interventions: Bonded Medical Places (access to a government subsidised university place) and Medical Rural Bonded Scholarships (scholarships paid to students during university training), [60]. Mathews et al. similarly did not differentiate between fellowships (funding provided to postgraduates training in a particular speciality), a bursaries program (scholarship to university students), and another program which provided special access to a postgraduate training place [50]. Hence it is not possible to determine which particular intervention(s) may have been associated with rural retention in these studies.

### Financial incentives

Five studies investigated associations between retention or turnover and various financial incentives, including having guaranteed access to paid locums [57], subsidized school fees for children [57], receiving retention incentive payments [35, 57], rural skills loading [57], increasing the salary of rural health professionals [34], reduced costs of malpractice insurance [61], and receiving Rural Doctors’ Association Settlement Package payments as a New South Wales (NSW) Visiting Medical Officer [37]. All studies involved doctors and took some account of potential confounders. One study was a simulation, another a discrete choice experiments and a further study recorded retention intentions [34, 57, 61]. Only two Australian studies entailed observed retention or turnover behaviour [35, 37].

Both a 50% increase in retention payments (*β* coefficient 1.423, *p* < 0.001) and 20% rural skills loadings (*β* coefficient 0.363, *p* < 0.001) were associated with increased probability of retaining General Practitioners (GPs), but were not as effective as providing guaranteed paid locum relief for 6 weeks every 12 months (*β* coefficient 1.624, *p* < 0.001) [57]. While locum relief incentives were important for retention of all rural GPs, regardless of location or on-call frequency, rural skills loadings were most important for GPs also doing hospital work. GPs with dependent children were also more responsive than GPs without dependent children to subsidised school fees [57]. Australian Government Rural Incentive Program payments were more effective in recruiting new GPs to incentivised rural areas rather than increasing the retention of existing GPs [35]. GP workforce retention was also significantly negatively associated with geographical remoteness in NSW, Australia, where GP retention incentives are scaled according to remoteness [37]. NSW GPs who were Visiting Medical Officers (and thereby received payments according to the Rural Doctors’ Association Settlement Package), had a 50% lower risk of leaving rural communities compared to GPs who were not [37]. In the USA, a 5% increase in rural county physician salary, simulated as an increase in reimbursement rate, was found to significantly decrease the probability of moving away from the same rural area; male physicians were more receptive to a policy change in reimbursement than their female counterparts [34]. Pepper et al. found that financial disincentives, in the form of high malpractice insurance rates, were associated with physicians planning to move their practice out of the (largely rural) state of Wyoming, rather than remaining in the state [61].

### Personal and professional support

Four studies examined the effectiveness of personal or professional support interventions on actual retention or turnover of rural health professionals [39–41, 55]. None adjusted for potential confounders. Offering medical students early sign-up to internships in a specific rural region, rather than going into a lottery for the opportunity to choose their preferred internship location, was associated with double the proportion of interns still working as physicians in the study area (29% versus 15%) [41]. However, retention of early sign-up interns was entirely in the most densely populated municipalities and none were recruited to any of the 15 remote municipalities. In a study of a cognitive behavioural coaching program (advertised as a work-life balance retreat), 94% of rural GPs who voluntarily attended were subsequently retained in rural general practice compared with 80% of the general rural doctor population (*p* = 0.027), despite their higher intention to leave rural general practice before coaching [40]. One study of an enhanced professional support network for rural surgeons added little to the extant literature as it lacked a comparator [39] and another low quality study reported that approximately two-thirds of survey respondents to a rural medical practice survey indicated that more reasonable hours of work, availability of locum tenens, availability of professional backup and educational opportunities for children would influence their retention intentions [55].

### Health systems

One USA study, where there is no universal health care insurance, used simulation to examine how expansion of publicly funded access to health care for some segments of the population (age 65 or older or younger but with disabilities—Medicare; low income—Medicaid) was associated with physician turnover [34]. Expansion of Medicaid and Medicare in rural areas was found to increase likelihood that physicians moved away from the rural county they worked in and became clinically inactive [34].

## Discussion

This review updates our understanding of the effectiveness of interventions to retain health workers in rural and remote areas. In contrast to a 2010 review which found little evidence of the effectiveness of any specific retention intervention except for regulatory interventions requiring ROS [17], this synthesis of evidence from 34 recent studies provides strong evidence about the effectiveness of educational interventions. Specifically, this review shows that a range of educational training pathway factors have strong associations with subsequent rural retention. These findings are consistent with other studies of associations between various rural pathway factors (not necessarily of interventions) and rural workforce supply (which reflect both recruitment and retention) [62, 63]. Policy makers can be confident that selecting rural (or remote) background students and training them in rural (or remote) areas, with the specific intention of preparing them for rural or remote practice, contributes to future rural retention.

Despite evidence from Northern Territory, Australia that Indigenous practitioners may have longer retention and lower turnover in remote communities [64], no study examined the effectiveness of selecting Indigenous students (or students from disadvantaged backgrounds) on subsequent retention in rural and remote Indigenous communities. The contribution of different elements of rural training programs to rural retention, such as the relative importance of mentorship by rural health professionals, rural career counselling and support and strong institutional social accountability mandates also remains unclear. Thus, associations between different elements of rural education pathways and retention remain largely untested and poorly understood.

Interventions requiring rural service in exchange for visa waivers or access to professional licenses or provider numbers were associated with comparatively low rural retention, especially once the ROS period was completed. Retention did, however, vary depending on the benefit accepted in exchange for rural service: health professionals choosing loan repayments tended to be more likely to stay following completion of ROS than health professionals accepting visa waivers, perhaps because recipients had greater choice in whether to enrol in the program than recipients seeking visa waivers. These findings were consistent with earlier US studies [65–68]. Current evidence therefore suggests that rural ROS programs which are strongly coercive should be used prudently if a primary aim of the program includes improved rural retention.

Evidence about the impact of financial incentives (with no ROS requirement) was limited because of the small number of studies and failure to quantify actual retention behaviour of health professionals. Perhaps the strongest evidence suggests that blanket financial retention incentives for rural GPs in Australia were ineffective [35]. This may partially be explained by the findings of another study which reported that many rural health professionals are not influenced by incentives of any type to stay, suggesting that any financial incentives should be tightly targeted only to those rural health professionals whose decisions about practice location are influenced by monetary incentives [57].

Despite these findings, the evidence-base could be stronger. The methodological quality of the studies was generally low. Only one-third of the studies were assessed as applying appropriate statistical analyses. Many studies lacked comparator groups or failed to account for potential confounders; others only provided a descriptive analysis. A further limitation was the heterogeneity of both interventions and study outcome measures, precluding a meta-analysis. All included studies were observational in design and thus subject to various types of bias—particularly selection bias—and unable to prove causality of associations. Definitions of rural and remote were not consistent between studies but were at the authors’ discretion. There was also an absence of cost-effectiveness studies. The systematic review method used also has its limitations, most especially its focus on what interventions work, without explicitly adequately investigating the contexts and mechanisms by which interventions are effective. This could be the subject of future research.

Most studies examined interventions for retaining doctors, with very few studies of nurses, Indigenous health practitioners or allied health professionals, thereby limiting the generalisability of the evidence to other health professions. Thus, despite the importance of comprehensive primary health care to improving the health outcomes for rural and remote populations, evidence on how best to retain nurses, Indigenous health practitioners and allied health professionals remains scant. This gap in the evidence, together with very few studies of retention in remote areas, may have a disproportionately negative impact on policy making in remote areas which frequently rely heavily on nurses, nurse practitioners and Indigenous health practitioners for primary health care service provision and where retention of health professionals can be extremely problematic [69].

## Conclusion

There is a growing body of evidence about the effectiveness of interventions to improve the retention of rural health professionals. The best available evidence suggests that policy makers can be confident that selecting health professional students based on rural background, encouraging distributed training based in rural and remote areas during their basic and subsequent training and removing barriers to rural health professionals for further developing their skills (both professional and personal) and qualifications is associated with longer rural retention.

However, there remain significant gaps in our knowledge and a stronger evidence base is required. Future research should seek to address methodological limitations, such as the lack of experimental studies and heterogeneity of retention outcome measures. The scope of future rural retention intervention studies should extend to include the retention of nurses, allied health professionals and Indigenous health practitioners, particularly in remote areas. These will lend greater confidence to policy makers to be able to justify and expand their armamentarium of potential interventions to improve retention and defray the high human and financial costs of rapid workforce turnover.

## Supplementary Information

**Additional file 1.** Medline search strategy.

**Additional file 2.** Studies excluded on full text.

**Additional file 3.** Quality appraisal of included studies.

## Data Availability

Not applicable.

## References

[1] Dolea C (2010). Increasing access to health workers in remote and rural areas through improved retention: global policy recommendations.

[2] Maarsingh OR (2016). Continuity of care in primary care and association with survival in older people: a 17-year prospective cohort study. Br J Gen Pract.

[3] Pereira-Gray DJ (2018). Continuity of care with doctors—a matter of life and death? A systematic review of continuity of care and mortality. BMJ Open.

[4] Australian Institute of Health and Welfare. Rural & remote health. Cat. no. PHE 255. AIHW: Canberra; 2019.

[5] Anderson TJ (2015). A cross-sectional study on health differences between rural and non-rural U.S. counties using the County Health Rankings. BMC Health Serv Res.

[6] Subedi R, Greenberg TL, Roshanafshar S (2019). Does geography matter in mortality? An analysis of potentially avoidable mortality by remoteness index in Canada. Health Rep.

[7] Tuttle C (2020). Rural-urban differences among older adults.

[8] Anderson I (2016). Indigenous and tribal peoples' health (The Lancet-Lowitja Institute Global Collaboration): a population study. Lancet.

[9] Schneider EC (2017). Mirror, Mirrow 2017: International comparison reflects flaws and opportunities for better U.S. health care.

[10] Humphreys JS (2007). Improving primary health care workforce retention in small rural and remote communities: how important is ongoing education and training?.

[11] Parlier AB (2018). The road to rural primary care: a narrative review of factors that help develop, recruit, and retain rural primary care Physicians. Acad Med.

[12] Campbell N, McAllister L, Eley D (2012). The influence of motivation in recruitment and retention of rural and remote allied health professionals: a literature review. Rural Remote Health.

[13] Rourke J (2010). WHO recommendations to improve retention of rural and remote health workers—important for all countries. Rural Remote Health.

[14] Wilson NW (2009). A critical review of interventions to redress the inequitable distribution of healthcare professionals to rural and remote areas. Rural Remote Health.

[15] Barnighausen T, Bloom DE (2009). Financial incentives for return of service in underserved areas: a systematic review. BMC Health Serv Res.

[16] Sempowski IP (2004). Effectiveness of financial incentives in exchange for rural and underserviced area return-of-service commitments: systematic review of the literature. Can J Rural Med.

[17] Buykx P (2010). Systematic review of effective retention incentives for health workers in rural and remote areas: towards evidence-based policy. Aust J Rural Health.

[18] Grobler L, et al. Interventions for increasing the proportion of health professionals practising in rural and other underserved areas. Cochrane Database Syst Rev. 2009(1). 10.1002/14651858.CD005314.pub2 19160251

[19] Moran AM (2014). Supervision, support and mentoring interventions for health practitioners in rural and remote contexts: an integrative review and thematic synthesis of the literature to identify mechanisms for successful outcomes. Hum Resour Health.

[20] Moola S, Aromataris E, Munn Z (2017). Chapter 7: Systematic reviews of etiology and risk. Joanna Briggs Institute reviewer’s manual.

[21] Hines S (2019). Retention strategies and interventions for health workers in rural and remote areas: a systematic review protocol. JBI Database Syst Rev Implement Rep.

[22] Holtom BC (2008). Turnover and retention research: a glance at the past, a closer review of the present, and a venture into the future. Acad Manag Ann.

[23] World Bank. World Bank country and lending groups. 2019. https://datahelpdesk.worldbank.org/knowledgebase/articles/906519-world-bank-country-and-lending-groups. Accessed 14 Jan 2021.

[24] Moher D (2009). Preferred reporting items for systematic reviews and meta-analyses: the PRISMA statement. J Clin Epidemiol.

[25] Aromataris E, Munn Z (2017). Joanna Briggs Institute reviewer's manual.

[26] World Health Organization (2010). Increasing access to health workers in remote and rural areas through improved retention—global policy recommendations.

[27] Putri LP (2020). Factors associated with increasing rural doctor supply in Asia-Pacific LMICs: a scoping review. Hum Resour Health.

[28] Murray MF (2011). The rural pipeline: building a strong nursing workforce through academic and service partnerships. Nurs Clin N Am.

[29] Nilsen G, Huemer J, Eriksen L (2012). Bachelor studies for nurses organised in rural contexts—a tool for improving the health care services in circumpolar region?. Int J Circumpolar Health.

[30] Norbye B, Skaalvik MW (2013). Decentralized nursing education in Northern Norway: towards a sustainable recruitment and retention model in rural Arctic healthcare services. Int J Circumpolar Health.

[31] Johnson G (2019). A longitudinal workforce analysis of a Rural Clinical Placement Program for final year dental students. Aust Dent J.

[32] Carson DB, Schoo A, Berggren P (2015). The 'rural pipeline' and retention of rural health professionals in Europe's northern peripheries. Health Policy.

[33] Renner DM (2010). The influence of loan repayment on rural healthcare provider recruitment and retention in Colorado. Rural Remote Health.

[34] Zhou JT (2018). Analyses of physician labor supply dynamics and its effect on patient welfare.

[35] Yong J (2018). Do rural incentives payments affect entries and exits of general practitioners?. Soc Sci Med.

[36] McGrail MR, Humphreys JS (2015). Geographical mobility of general practitioners in rural Australia. Med J Aust.

[37] Russell DJ (2013). The value of survival analyses for evidence-based rural medical workforce planning. Hum Resour Health.

[38] Kwan MMS (2017). The rural pipeline to longer-term rural practice: general practitioners and specialists. PLoS ONE.

[39] Cogbill TH, Bintz M (2017). Rural general surgery: a 38-year experience with a regional network established by an integrated health system in the Midwestern United States. J Am Coll Surg.

[40] Gardiner M, Kearns H, Tiggemann M (2013). Effectiveness of cognitive behavioural coaching in improving the well-being and retention of rural general practitioners. Aust J Rural Health.

[41] Gaski M, Abelsen B (2017). Designing medical internships to improve recruitment and retention of doctors in rural areas. Int J Circumpolar Health.

[42] Gorsche RG, Woloschuk W (2012). Rural physicians' skills enrichment program: a cohort control study of retention in Alberta. Aust J Rural Health.

[43] Rabinowitz HK (2013). Retention of rural family physicians after 20–25 years: outcomes of a comprehensive medical school rural program. J Am Board Fam Med.

[44] Robinson M, Slaney GM (2013). Choice or chance! The influence of decentralised training on GP retention in the Bogong region of Victoria and New South Wales. Rural Remote Health.

[45] Rodney WM (2010). OB fellowship outcomes 1992–2010: where do they go, who stops delivering, and why?. Fam Med.

[46] Ross R (2013). Fifteen-year outcomes of a rural residency: aligning policy with national needs. Fam Med.

[47] Wearne S (2010). Where are they now? The career paths of the Remote Vocational Training Scheme registrars. Aust Fam Physician.

[48] Fleming P, Mathews M (2012). Retention of specialist physicians in Newfoundland and Labrador. Open Med.

[49] Kahn TR, Hagopian A, Johnson K (2010). Retention of J-1 visa waiver program physicians in Washington State's health professional shortage areas. Acad Med.

[50] Mathews M (2013). Evaluation of physician return-for-service agreements in Newfoundland and Labrador. Healthc Policy.

[51] Opoku ST (2015). A comparison of the J-1 visa waiver and loan repayment programs in the recruitment and retention of physicians in rural Nebraska. J Rural Health.

[52] Straume K, Søndenå MS, Prydz P (2010). Postgraduate training at the ends of the earth—a way to retain physicians?. Rural Remote Health.

[53] Jamieson JL (2013). One program, multiple training sites: does site of family medicine training influence professional practice location?. Rural Remote Health.

[54] Woolley T, Sen Gupta T, Bellei M (2017). Predictors of remote practice location in the first seven cohorts of James Cook University MBBS graduates. Rural Remote Health.

[55] Chauhan TS, Jong M, Buske L (2010). Recruitment trumps retention: results of the 2008/09 CMA Rural Practice Survey. Can J Rural Med.

[56] Jamar E, Newbury J, Mills D (2014). Early career location of University of Adelaide rural cohort medical students. Rural Remote Health.

[57] Li J (2014). Retaining rural doctors: doctors' preferences for rural medical workforce incentives. Soc Sci Med.

[58] McGrail MR, Russell DJ, Campbell DG (2016). Vocational training of general practitioners in rural locations is critical for the Australian rural medical workforce. Med J Aust.

[59] Patterson DG (2016). Family medicine rural training track residencies: 2008–2015 graduate outcomes.

[60] Playford D (2019). Graduate doctors' rural work increases over time. Med Teach.

[61] Pepper CM, Sandefer RH, Gray MJ (2010). Recruiting and retaining physicians in very rural areas. J Rural Health.

[62] Ranmuthugala G (2007). Where is the evidence that rural exposure increases uptake of rural medical practice?. Aust J Rural Health.

[63] Greenhill JA, Walker J, Playford D (2015). Outcomes of Australian rural clinical schools: a decade of success building the rural medical workforce through the education and training continuum. Rural Remote Health.

[64] Wakerman J (2019). Remote health workforce turnover and retention: what are the policy and practice priorities?. Hum Resour Health.

[65] Rosenblatt RA (1996). Beyond retention: National Health Service Corps participation and subsequent practice locations of a cohort of rural family physicians. J Am Board Fam Pract.

[66] Pathman DE, Konrad TR, Ricketts TC (1992). The comparative retention of National Health Service Corps and other rural physicians. Results of a 9-year follow-up study. JAMA J Am Med Assoc.

[67] Lapolla M (2004). State public policy: the impacts of Oklahoma's physician incentive programs. J Okla State Med Assoc.

[68] Crouse BJ, Munson RL (2006). The effect of the physician J-1 visa waiver on rural Wisconsin. Wis Med J.

[69] Russell DJ, et al. Health workforce turnover, stability and employment survival in remote NT health centres 2004–15. In: Proceedings of the 15th national rural health conference, 24–27 March, 2019. Hobart, Tasmania: National Rural Health Alliance.

