# Supplementary material for: Interventions for health workforce retention in rural and remote areas: a systematic review
Source: Hum Resour Health. 2021 Aug 26;19:103. doi: 10.1186/s12960-021-00643-7 (PMC8393462; doi:10.1186/s12960-021-00643-7)
Supplement: Supplementary file 1 — Additional file 1. Medline search strategy. [file 12960_2021_643_MOESM1_ESM.docx]

## Additional file 1: Medline search strategy

1 exp Health Personnel/

2 Health Manpower/

3 manpower.fs.

4 nurse?.tw.

5 (midwives or mid wives or midwife or mid wife).tw.

6 (physician? or doctor? or practitioner? or GP?).tw.

7 dentist?.tw.

8 dental staff.tw.

9 pharmacist$.tw.

10 dietician?.tw.

11 nutritionist?.tw.

12 psychologist?.tw.

13 occupational therapist?.tw.

14 (physiotherapist? or physical therapist?).tw.

15 language therapist?.tw.

16 speech therapist?.tw.

17 (logopaedist? or logopedist?).tw.

18 speech pathologist?.tw.

19 language pathologist?.tw.

20 audiologist?.tw.

21 internist?.tw.

22 (paediatrician? or pediatrician?).tw.

23 opthalmologist?.tw.

24 surgeon?.tw.

25 (radiographer? or radiologist?).tw.

26 optometrist?.tw.

27 ((health or healthcare or health care) adj (personnel or worker? or staff or professional? or provider?)).tw.

28 (medical adj (personnel or staff or professional? or worker?)).tw.

29 (nurs$ adj (personnel or staff or professional? or worker?)).tw.

30 (health manpower or human resources or workforce?).tw.

31 exp Personnel Management/

32 Resource Allocation/

33 Personnel Turnover/

34 (((client adj1 staff) or (patient adj1 staff) or (patient adj1 nurse)) adj ratio).tw.

35 ((physician? or doctor? or practitioner? or nurse? or personnel or staff or professional?) adj2 shortage).tw.

36 understaff$.tw.

37 ((personnel or staff) adj (recruitment or retain$ or retention or turnover or turn over)).tw.

38 "Personnel Staffing and Scheduling"/og

39 or/1-38

40 Rural Health Services/

41 Medically Underserved Area/

42 Hospitals, Rural/

43 ((rural or remote or nonmetropolitan) adj (health service? or health care or healthcare or medical service? or medical care)).tw.

44 (rural adj (setting? or clinic? or hospital?)).tw.

45 shortage area?.tw.

46 (inequitable distribut$ or maldistribut$).tw.

47 Professional Practice Location/

48 PHYSICIANS/sd

49 Rural Health/ma

50 ((rural or remote or nonmetropolitan or underserved or under served or deprived) adj (communit$ or area? or region? or province?)).tw.

51 or/40-50

52 (((((Andorra or Germany or Oman or Antigua) and Barbuda) or Gibraltar or Palau or Argentina or Greece or Panama or Aruba or Greenland or Poland or Australia or Guam or Portugal or Austria or Hong Kong or Puerto Rico or Bahamas or Hungary or Qatar or Bahrain or Iceland or San Marino or Barbados or Ireland or Saudi Arabia or Belgium or Isle of Man or Seychelles or Bermuda or Israel or Singapore or British Virgin Islands or Italy or Sint Maarten or Brunei Darussalam or Japan or Slovak Republic or Canada or Korea or Slovenia or Cayman Islands or Kuwait or Spain or Channel Islands or Latvia or (St Kitts and Nevis) or Chile or Liechtenstein or St Martin or Croatia or Lithuania or Sweden or Curacao or Luxembourg or Switzerland or Cyprus or Macao or Taiwan or Czech Republic or Malta or Trinidad) and Tobago) or Denmark or Monaco or (Turks and Caicos Islands) or Estonia or Netherlands or United Arab Emirates or Faroe Islands or New Caledonia or United Kingdom or Finland or New Zealand or United States or France or Northern Mariana Islands or Uruguay or French Polynesia or Norway or Virgin Islands).mp. [mp=title, abstract, original title, name of substance word, subject heading word, floating sub-heading word, keyword heading word, protocol supplementary concept word, rare disease supplementary concept word, unique identifier, synonyms]

53 Rural Population/

54 52 or 53

55 incentive.mp. or Motivation/

56 reward.mp. or REWARD/

57 financial incentive.mp. or Physician Incentive Plans/

58 (monetary incentive or non-financial incentive or non-monetary incentive or allowances or salaries or benefits; compulsory service or bonding scheme; rural pipeline or professional development or professional support or health vacancy rates or motivation).mp.

59 (recruit? or retain?).mp. or retention.mp

60 or/55-59

61 39 and 51 and 54 and 60
