# Supplementary material for: Interventions for health workforce retention in rural and remote areas: a systematic review
Source: Hum Resour Health. 2021 Aug 26;19:103. doi: 10.1186/s12960-021-00643-7 (PMC8393462; doi:10.1186/s12960-021-00643-7)
Supplement: Supplementary file 2 — Additional file 2. Studies excluded on full text. [file 12960_2021_643_MOESM2_ESM.docx]

## Additional file 2: Studies excluded on full text

Agiro A, Wan TTH, Ortiz J. Organizational and Environmental Correlates to Preventive Quality of Care in US Rural Health Clinics. Journal of primary care & community health. 2012;3(4):264–71.

Reason for exclusion: outcome not rural or remote retention

Aiga H. Train to retain or drain?. The need for a global survey for sitting allowances. Public Health. 2012;126(7):620–3.

Reason for exclusion: study type

Ampofo-Addo O, Mou H, Olfert R, Goodridge D. Location decisions of family physicians in Saskatchewan: What really matters? Canadian journal of rural medicine : the official journal of the Society of Rural Physicians of Canada = Journal canadien de la medecine rurale : le journal officiel de la Societe de medecine rurale du Canada. 2016;21(1):7–12.

Reason for exclusion: outcome not rural or remote retention

Anonymous. Management Updates: Staff Passion: Your Script for Success. The Australian Journal of Pharmacy. 2010;91(1086):60–1.

Reason for exclusion: study type

Anthony D, El Rayess F, Esquibel AY, George P, Taylor J. Building a workforce of physicians to care for underserved patients. Rhode Island medical journal (2013). 2014;97(9):31–5.

Reason for exclusion: outcome not rural or remote retention

Bacopanos E, Edgar S. Employment patterns of Notre Dame graduate physiotherapists 2006-12: Targeting areas of workforce need. Australian Health Review. 2016;40(2):188–93.

Reason for exclusion: outcome not rural or remote retention

Bailey BE, Wharton RG, Holman CD. Glass half full: Survival analysis of new rural doctor retention in Western Australia. Aust J Rural Health. 2016;24(4):258–64.

Reason for exclusion: no intervention

Balasubramanian M, Teusner DN. Dental statistics and research series no 53. In: Dentists, specialists and allied practitioners in Australia: Dental Labour Force Collection. Dentists, Specialists and Allied Practitioners in Australia: Dental Labour Force Collection, 2006. 2011;

Reason for exclusion: outcome not rural or remote retention

Bentz BB. Retention of International Medical Graduates Participating in the Iowa Conrad 30 Program. 2018. p. 248.

Reason for exclusion: not rural setting

Bhatavadekar NB, Rozier RG, Konrad TR. Holding up the oral health safety net: the role of National Health Service Corps alumni dentists in North Carolina. International dental journal. 2011;61(3):136–43.

Reason for exclusion: not rural setting

Briggs B. Solutions for recruitment and retention of rural psychologists by rural psychologists. 2015. p. 85.

Reason for exclusion: outcome not rural or remote retention

Brown L, Williams L, Capra S. Going rural but not staying long: Recruitment and retention issues for the rural dietetic workforce in Australia. Nutrition & Dietetics. 2010;67(4):294–302.

Reason for exclusion: study type

Canadian Institute for Health Information. Occupational Therapists in Canada, National Jurisdictional Highlights and Profiles. 2011;

Reason for exclusion: outcome not rural or remote retention

Canadian Institute for Health Information. Physiotherapists in Canada, 2010 National and Jurisdictional Highlights and Profiles. Physiotherapy Database. 2011;

Reason for exclusion: outcome not rural or remote retention

Ceramidas DM. A case against generalisation of mental health occupational therapy in Australia. Australian occupational therapy journal. 2010;57(6):409–16.

Reason for exclusion: outcome not rural or remote retention

Cho SH, Lee JY, Mark BA, Jones CB. Geographic mobility of Korean new graduate nurses from their first to subsequent jobs and metropolitan-nonmetropolitan differences in their job satisfaction. Nursing Outlook. 2014;62(1):22–8.

Reason for exclusion: no intervention

Cho SH, Lee JY, Mark BA, Lee HY. Geographical imbalances: migration patterns of new graduate nurses and factors related to working in non-metropolitan hospitals. J Korean Acad Nurs. 2012;42(7):1019–26.

Reason for exclusion: outcome not rural or remote retention

Collier D. Rural Michigan physician retention study reveals motivators. Michigan medicine. 2010;109(5):21.

Reason for exclusion: study type

Crane S, Jones G. Innovation in rural family medicine training: the Mountain Area Health Education Center’s rural-track residency program. North Carolina medical journal. 2014;75(1):29–30.

Reason for exclusion: study type

Deutchman M. Medical School Rural Tracks in the US. Policy Brief. 2013;

Reason for exclusion: outcome not rural or remote retention

Devine SG, Williams G, Nielsen I. Rural Allied Health Scholarships: do they make a difference? Rural and remote health. 2013;13(4):2459.

Reason for exclusion: outcome not rural or remote retention

Duffrin C, Diaz S, Cashion M, Watson R, Cummings D, Jackson N. Factors associated with placement of rural primary care physicians in North Carolina. Southern medical journal. 2014;107(11):728–33.

Reason for exclusion: outcome not rural or remote retention

Dyck KG, Hardy C. Enhancing access to psychologically informed mental health services in rural and northern communities. Canadian Psychology. 2013;54(1):30–7.

Reason for exclusion: study type

Eley DS, Synnott R, Baker PG, Chater AB. A decade of Australian Rural Clinical School graduates - where are they and why? Rural and remote health. 2012;12(1).

Reason for exclusion: outcome not rural or remote retention

Fagan EB, Gibbons C, Finnegan SC, Petterson S, Peterson LE, Phillips RL, et al. Family medicine graduate proximity to their site of training: policy options for improving the distribution of primary care access. Family Medicine. 2015;47(2):124–30.

Reason for exclusion: not rural setting

Ferguson-Paré M, Mallette C, Zarins B, McLeod S, Reuben K. Collaboration to change the landscape of nursing: a journey between urban and remote practice settings. Nursing leadership (Toronto, Ont). 2010;23 Spec No 2010:90–100.

Reason for exclusion: outcome not rural or remote retention

Filipova AA. Factors influencing the satisfaction of rural physician assistants: a cross-sectional study. Journal of allied health. 2014;43(1):22–31.

Reason for exclusion: no intervention

Fryer S. Senate Community Affairs Committee Submission: Inquiry into the Factors Affecting the Supply of Health Services and Medical Professionals in Rural Areas. 2012.

Reason for exclusion: full text not available

Gallego G, Dew A, Lincoln M, Bundy A, Chedid RJ, Bulkeley K, et al. Should I stay or should I go? Exploring the job preferences of allied health professionals working with people with disability in rural Australia. Human Resources for Health. 2015;13:53.

Reason for exclusion: no intervention

Gupta S, Ngo H, Burkitt T, Puddey I, Playford D. Survival analysis of Rural Clinical School of Western Australia graduates: the long-term work of building a long-term rural medical workforce. BMC Health Serv Res. 2019;19(1):998.

Reason for exclusion: Doesn't add any useful data, duplicate cohort

Healey-Ogden M, Wejr P, Farrow C. British Columbia: improving retention and recruitment in smaller communities. Nursing leadership (Toronto, Ont). 2012;25 Spec No 2012:37–44.

Reason for exclusion: study type

Herd MS, Bulsara MK, Jones MP, Mak DB. Preferred practice location at medical school commencement strongly determines graduates’ rural preferences and work locations. Australian Journal of Rural Health. 2017;25(1):15–21.

Reason for exclusion: outcome not rural or remote retention

Hill ME, Raftis D, Wakewich P. Strengthening the rural dietetics workforce: Examining early effects of the Northern Ontario Dietetic Internship Program on recruitment and retention. Rural and remote health. 2017;17(1).

Reason for exclusion: outcome not rural or remote retention

Hirsh D, Walters L, Poncelet AN. Better learning, better doctors, better delivery system: Possibilities from a case study of longitudinal integrated clerkships. Medical Teacher. 2012;34(7):548–54.

Reason for exclusion: outcome not rural or remote retention

Humphreys JS, Chisholm MC, Russell DJ. Rural allied health workforce retention in Victoria: modelling the benefits of increased length of stay and reduced staff turnover. Final Report for Victorian Department of Health, Workforce Innovation Grant Program 2008-2009. 2010.

Reason for exclusion: no intervention

Insync surveys. The 2012 Insync Surveys Retention Review. 2012;

Reason for exclusion: not rural setting

Johnson DS. Exploring Barriers and Resources to Train and Retain PMHNPs in a Rural Community. 2017. p. 107.

Reason for exclusion: outcome not rural or remote retention

Jones D. GP recruitment and retention. British Journal of General Practice. 2015;65(634):230.

Reason for exclusion: study type

Jones MP, Humphreys JS, Nicholson T. Is personality the missing link in understanding recruitment and retention of rural general practitioners? Australian Journal of Rural Health. 2012;20(2):74–9.

Reason for exclusion: no intervention

Joyce CM, Schurer S, Scott A, Humphreys J, Kalb G. Australian doctors’ satisfaction with their work: results from the MABEL longitudinal survey of doctors. Med J Aust. 2011;194(1):30–3.

Reason for exclusion: outcome not rural or remote retention

Kataoka Y, Takayashiki A, Sato M, Maeno T. Japanese regional-quota medical students in their final year are less motivated to work in medically underserved areas than they were in their first year: A prospective observational study. Rural and remote health. 2018;18(4).

Reason for exclusion: participant type

Keane S, Lincoln M, Rolfe M, Smith T. Retention of the rural allied health workforce in New South Wales: A comparison of public and private practitioners. BMC Health Services Research. 2013;13:32.

Reason for exclusion: no intervention

Keane S, Smith T, Lincoln M, Fisher K. Survey of the rural allied health workforce in New South Wales to inform recruitment and retention. Aust J Rural Health. 2011;19(1):38–44.

Reason for exclusion: no intervention

Kirkman JM, Bentley SA, Armitage JA, Woods CA. Could adoption of the rural pipeline concept redress Australian optometry workforce issues? Clinical and Experimental Optometry. 2019;

Reason for exclusion: study type

Kovner CT, Corcoran SP, Brewer CS. The relative geographic immobility of new registered nurses calls for new strategies to augment that workforce. Health affairs (Project Hope). 2011;30(12):2293–300.

Reason for exclusion: outcome not rural or remote retention

Kulig JC, Kilpatrick K, Moffitt P, Zimmer L. Recruitment and Retention in Rural Nursing: It’s Still an Issue! Nursing leadership (Toronto, Ont). 2015;28(2):40–50.

Reason for exclusion: study type

Kutscher B. The rural route. Hospitals in underserved areas taking different roads to recruit, retain physicians. Modern healthcare. 2013;43(18):30–1.

Reason for exclusion: study type

Larson L. “Time Off Pays Off.” Hospitals & health networks. 2014;

Reason for exclusion: study type

Leibert M, Leaming LE. Critical access hospital chief executive officer turnover: implications and challenges for governing boards. The health care manager. 2010;29(1):22–8.

Reason for exclusion: study type

Lowen T. The hunt for new recruits. Employers are working harder to get and keep primary care physicians. Minnesota medicine. 2013;96(2):18–21.

Reason for exclusion: study type

Luzzi L, Spencer AJ. Job satisfaction of the oral health labour force in Australia. Aust Dent J. 2011;56(1):23–32.

Reason for exclusion: outcome not rural or remote retention

MacVicar R, Clarke G, Hogg DR. Scotland’s GP rural fellowship: An initiative that has impacted on rural recruitment and retention. Rural and remote health. 2016;16(1).

Reason for exclusion: outcome not rural or remote retention

Mathews M, Ryan D. Financial recruitment incentive programs for dentists in newfoundland and labrador. Journal of the Canadian Dental Association. 2014;80.

Reason for exclusion: outcome not rural or remote retention

Matsumoto M, Inoue K, Kajii E. Policy implications of a financial incentive programme to retain a physician workforce in underserved Japanese rural areas. Social Science and Medicine. 2010;71(4):667–71.

Reason for exclusion: outcome not rural or remote retention

Mazumdar S, McRae I. Doctors on the move: National estimates of geographical mobility among general practitioners in Australia. Australian Family Physician. 2015;44(10):747–51.

Reason for exclusion: no intervention

McFarland KK, Reinhardt JW, Yaseen M. Rural dentists: does growing up in a small community matter? Journal of the American Dental Association (1939). 2012;143(9):1013–9.

Reason for exclusion: outcome not rural or remote retention

McGrail MR, Humphreys JS, Scott A, Joyce CM, Kalb G. Professional satisfaction in general practice: Does it vary by size of community? Medical Journal of Australia. 2010;193(2):94–8.

Reason for exclusion: outcome not rural or remote retention

McGrail MR, Russell DJ, O’Sullivan BG. Family effects on the rurality of GP’s work location: a longitudinal panel study. Human Resources for Health. 2017;15.

Reason for exclusion: no intervention

McGrail MR, Wingrove PM, Petterson SM, Bazemore AW. Mobility of US Rural Primary Care Physicians During 2000-2014. Annals of family medicine. 2017;15(4):322–8.

Reason for exclusion: no intervention

McKillop A, Webster C, Bennett W, O’Connor B, Bagg W. Encouragers and discouragers affecting medical graduates’ choice of regional and rural practice locations. Rural and remote health. 2017;17(4):4247.

Reason for exclusion: outcome not rural or remote retention

Morell AL, Kiem S, Millsteed MA, Pollice A. Attraction, recruitment and distribution of health professionals in rural and remote Australia: early results of the Rural Health Professionals Program. Human Resources for Health. 2014;12:15.

Reason for exclusion: outcome not rural or remote retention

Murphy F. Remote incentives for nurses. Nursing Review. 2013;

Reason for exclusion: outcome not rural or remote retention

Myroniuk L, Adamiak P, Bajaj S, Myhre DL. Recruitment and retention of physicians in rural Alberta: the spousal perspective. Rural Remote Health. 2016;16(1):3620.

Reason for exclusion: no intervention

National Rural Health Student Network. Bonded medical places and medical rural bonded scholarships: position paper. Melbourne. 2010;

Reason for exclusion: outcome not rural or remote retention

Nelson GC, Gruca TS. Determinants of the 5-Year Retention and Rural Location of Family Physicians: Results from the Iowa Family Medicine Training Network. Fam Med. 2017;49(6):473–6.

Reason for exclusion: not rural setting

Nelson M, Bunyard J, Quinn S, Williams D. Porrige: A cohort study of general practice registrars. Australian Family Physician. 2011;40(3):138–41.

Reason for exclusion: outcome not rural or remote retention

Nojima Y, Kumakura S, Onoda K, Hamano T, Kimura K. Job and life satisfaction and preference of future practice locations of physicians on remote islands in Japan. Human Resources for Health. 2015;13:39.

Reason for exclusion: outcome not rural or remote retention

Onnis LL. An examination of supportive management practices promoting health workforce stability in remote northern Australia. Australasian Psychiatry. 2015;23(6):679–82.

Reason for exclusion: outcome not rural or remote retention

Opie T, Dollard M, Lenthall S, Wakerman J, Dunn S, Knight S, et al. Levels of occupational stress in the remote area nursing workforce. Australian Journal of Rural Health. 2010;18(6):235–41.

Reason for exclusion: outcome not rural or remote retention

Opie T, Lenthall S, Wakerman J, Dollard M, MacLeod M, Knight S, et al. Occupational stress in the Australian nursing workforce: A comparison between hospital-based nurses and nurses working in very remote communities. Australian Journal of Advanced Nursing. 2011;28(4):36–43.

Reason for exclusion: outcome not rural or remote retention

Orda U, Orda S, Gupta TS, Knight S. Building a sustainable workforce in a rural and remote health service: A comprehensive and innovative Rural Generalist training approach. Australian Journal of Rural Health. 2017;25(2):116–9.

Reason for exclusion: outcome not rural or remote retention

O’Dowd A. New GPs are offered 20,000 to work in underserved areas. BMJ (Clinical research ed). 2016;352:i729.

Reason for exclusion: outcome not rural or remote retention

O’Sullivan B, McGrail M, Russell D. Rural specialists: The nature of their work and professional satisfaction by geographical location of work. Australian Journal of Rural Health. 2017;25(6):338–46.

Reason for exclusion: outcome not rural or remote retention

O’Toole K, Schoo AM. Retention policies for allied health professionals in rural areas: a survey of private practitioners. Rural and remote health. 2010;10(2):1331.

Reason for exclusion: outcome not rural or remote retention

Patterson DG, Longenecker R, Schmitz D, Phillips RL, Skillman SM, Doescher MP. Rural Residency Training for Family Medicine Physicians: Graduate Early-Career Outcomes, 2008-2012. 2013;

Reason for exclusion: Duplicate cohort

Pena S, Ramirez J, Becerra C, Carabantes J, Arteaga O. The Chilean Rural Practitioner Programme: a multidimensional strategy to attract and retain doctors in rural areas. Bulletin of the World Health Organization. 2010;88(5):371–8.

Reason for exclusion: not high income

Playford D, Wheatland B, Larson A. Does teaching an entire nursing degree rurally have more workforce impact than rural placements? Contemporary Nurse. 2010;35(1):068–76.

Reason for exclusion: outcome not rural or remote retention

Playford DE, Cheong E. Rural Undergraduate Support and Coordination, Rural Clinical School, and Rural Australian Medical Undergraduate Scholarship: rural undergraduate initiatives and subsequent rural medical workforce. Australian Health Review. 2012;36(3):301–7.

Reason for exclusion: outcome not rural or remote retention

Playford DE, Ng WQ, Burkitt T. Creation of a mobile rural workforce following undergraduate longitudinal rural immersion. Medical Teacher. 2016;38(5):498–503.

Reason for exclusion: Doesn't add any useful data, duplicate cohort

Pugh JD, Twigg DE, Martin TL, Rai T. Western Australia facing critical losses in its midwifery workforce: A survey of midwives’ intentions. Midwifery. 2013;29(5):497–505.

Reason for exclusion: not rural setting

Ramos P, Alves H. Doctors’ career choices in health systems constrained by national medical exams: A discrete choice experiment. The International journal of health planning and management. 2018;33(4):e1211–24.

Reason for exclusion: outcome not rural or remote retention

Ranmuthugala G. Rural recruitment and training promotes rural practice by GPs, but is it enough to retain them? The Medical journal of Australia. 2016;205(5):210–1.

Reason for exclusion: study type

Reardon TF. Nursing recruitment and retention issues: similarities and differences rural United States and Australia. 2010. p. 150 p-150 p.

Reason for exclusion: no intervention

Rural Health Education Development (Rh Ed) Consulting Pty Ltd. Research to Track the Rural Pharmacy Workforce and Identify the Role that Rural Programs have on Retention of the Rural Pharmacy Workforce. 2010.

Reason for exclusion: outcome not rural or remote retention

Rockey PH, Rieselbach RE, Neuhausen K. States can transform their health care workforce. J Grad Med Educ. 2014;6:805–8.

Reason for exclusion: outcome not rural or remote retention

Roots A, MacDonald M. Outcomes associated with nurse practitioners in collaborative practice with general practitioners in rural settings in Canada: a mixed methods study. Human Resources for Health. 2014;12(1):69–69.

Reason for exclusion: study type

Rourke J, Asghari S, Hurley O, Ravalia M, Jong M, Parsons W, et al. From pipelines to pathways: the Memorial experience in educating doctors for rural generalist practice. Rural and remote health. 2018;18(1):4427.

Reason for exclusion: outcome not rural or remote retention

Russell DJ, McGrail MR, Humphreys JS, Wakerman J. What factors contribute most to the retention of general practitioners in rural and remote areas? Australian Journal of Primary Health. 2012;18(4):289–94.

Reason for exclusion: no intervention

Russell DJ, Wakerman J, Humphreys JS. What is a reasonable length of employment for health workers in Australian rural and remote primary healthcare services? Australian Health Review. 2013;37(2):256–61.

Reason for exclusion: no intervention

Russell DJ, Wakerman J, Humphreys JS. What is a reasonable length of employment for health workers in Australian rural and remote primary healthcare services? Australian Health Review. 2013;37(2):256–61.

Reason for exclusion: duplicate cohort

Russell DJ, Zhao Y, Guthridge S, Ramjan M, Jones MP, Humphreys JS, et al. Patterns of resident health workforce turnover and retention in remote communities of the Northern Territory of Australia, 2013–2015. Human Resources for Health. 2017;15(1):52.

Reason for exclusion: no intervention

Russell DJ, Zhao Y, Guthridge S, Wright J, Ramjan M, Jones MP, et al. Health workforce turnover, stability and employment survival in remote NT health centres 2004-15. In: Proceedings of the 15th National Rural Health Conference, 24-27 March. 2019.

Reason for exclusion: no intervention

Sen Gupta T, Woolley T, Murray R, Hays R, McCloskey T. Positive impacts on rural and regional workforce from the first seven cohorts of James Cook University medical graduates. Rural and Remote Health. 2014;14:2657.

Reason for exclusion: Duplicate cohort

Seymour MW. Registered nurses working in critical access hospitals and their perceptions of psychological and structural empowerment. 2015. p. 121.

Reason for exclusion: no intervention

Slagle DR. Rural versus urban: Tennessee health administrators’ strategies on recruitment and retention for allied health professionals. 2010. p. 163.

Reason for exclusion: outcome not rural or remote retention

Smedts AM, Campbell N, Sweet L. Work-integrated learning (WIL) supervisors and nonsupervisors of allied health professional students. Rural and remote health. 2013;13(1).

Reason for exclusion: no intervention

Smith JD, White C, Roufeil L, Veitch C, Pont L, Patel B, et al. A national study into the rural and remote pharmacist workforce. Rural Remote Health. 2013;13(2):2214.

Reason for exclusion: outcome not rural or remote retention

Smith T, Fisher K, Keane S, Lincoln M. Comparison of the results of two rural allied health workforce surveys in the Hunter New England region of New South Wales: 2005 versus 2008. The Australian journal of rural health. 2011;19(3):154–9.

Reason for exclusion: no intervention

Straume K, Shaw DMP. Effective physician retention strategies in Norway’s northernmost county. Bulletin of the World Health Organization. 2010;88(5):390–4.

Reason for exclusion: Duplicate cohort

Straume K, Shaw DMP. Internship at the ends of the earth - a way to recruit physicians? Rural and remote health. 2010;10(2):1366.

Reason for exclusion: outcome not rural or remote retention

Teixeira-Poit SM, Halpern MT, Kane HL, Keating M, Olmsted M. Factors influencing professional life satisfaction among neurologists. BMC health services research. 2017;17(1):409.

Reason for exclusion: no intervention

Terry DR, Lê Q, Hoang H. Satisfaction amid professional challenges: International medical graduates in rural tasmania. Australasian Medical Journal. 2014;7(12):500–17.

Reason for exclusion: no intervention

Thoms D. Rural health: it’s time to address the issues. Nursing Review. 2013;(4):36–7.

Reason for exclusion: study type

Tyrrell MS, Carey TA, Wakerman J. The work motivations of the health practitioner who stays for a substantial time in the very remote Indigenous community workplace. Australian Journal of Psychology. 2018;70(4):318–29.

Reason for exclusion: no intervention

Whitford D, Smith T, Newbury J. The South Australian Allied Health Workforce survey: helping to fill the evidence gap in primary health workforce planning. Aust J Prim Health. 2012;18(3):234–41.

Reason for exclusion: no intervention

Wielandt PM, Taylor E. Understanding rural practice: implications for occupational therapy education in Canada. Rural & Remote Health. 2010;10(3):1–13.

Reason for exclusion: no intervention

Witt J. Physician recruitment and retention in Manitoba: results from a survey of physicians’ preferences for rural jobs. Canadian journal of rural medicine : the official journal of the Society of Rural Physicians of Canada = Journal canadien de la medecine rurale : le journal officiel de la Societe de medecine rurale du Canada. 2017;22(2):43–53.

Reason for exclusion: no intervention

Xierali IM, Sweeney SA, Phillips Jr RL, Bazemore AW, Petterson SM. Increasing Graduate Medical Education (GME) in Critical Access Hospitals (CAH) could enhance physician recruitment and retention in rural America. Journal of the American Board of Family Medicine. 2012;25(1):7–8.

Reason for exclusion: study type
