# Supplementary material for: Interventions for health workforce retention in rural and remote areas: a systematic review
Source: Hum Resour Health. 2021 Aug 26;19:103. doi: 10.1186/s12960-021-00643-7 (PMC8393462; doi:10.1186/s12960-021-00643-7)
Supplement: Supplementary file 3 — Additional file 3. Quality appraisal of included studies. [file 12960_2021_643_MOESM3_ESM.docx]

## Additional file 3: Quality appraisal of included studies

| Citation | Study type | Q1 | Q2 | Q3 | Q4 | Q5 | Q6 | Q7 | Q8 | Q9 | Q10 | Q11 | Score |
| --- | --- | --- | --- | --- | --- | --- | --- | --- | --- | --- | --- | --- | --- |
| Carson *et* al. (2015) [32] | cross-sectional | U | N | N | N/A | Y | Y | U | N | - | - | - | 6/16 |
| Chauhan *et* al. (2010) [55] | cross-sectional | Y | N | U | N/A | N | N/A | U | N | - | - | - | 4/16 |
| Cogbill & Bintz. (2017) [39] | case report | Y | U | Y | Y | N | U | N | U | - | - | - | 9/16 |
| Fleming & Mathews. (2012) [48] | cohort | Y | Y | Y | Y | Y | Y | Y | Y | y | Y | Y | 22/22 |
| Gardiner *et* al. (2013) [40] | cohort | Y | Y | Y | N | N/A | N | Y | Y | Y | Y | N | 14/22 |
| Gaski & Abelsen. (2017) [41] | cohort | Y | Y | Y | N | N/A | Y | Y | Y | Y | Y | N | 16/22 |
| Gorsche & Woloschuk. (2012) [42] | cohort | Y | Y | Y | Y | Y | Y | Y | Y | N | N | N | 16/22 |
| Jamar *et* al. (2014) [56] | cohort | N/A | N/A | U | Y | N | Y | N | N | N | N | N | 5/22 |
| Jamieson *et* al. (2013) [53] | cohort | Y | Y | Y | Y | Y | U | Y | Y | N | Y | Y | 19/22 |
| Johnson et al. (2019) [31] | cohort | Y | Y | Y | Y | Y | Y | Y | Y | Y | Y | Y | 22/22 |
| Kahn *et* al. (2010) [49] | cohort | N/A | N/A | Y | Y | N | Y | Y | U | N | U | N | 10/22 |
| Kwan *et al*. (2017) [38] | cohort | U | Y | Y | Y | Y | Y | Y | N | N | U | Y | 16/22 |
| Li *et* al. (2014) [57] | cross-sectional and discrete choice experiment | Y | Y | Y | N/A | Y | Y | Y | Y | - | - | - | 14/16 |
| Mathews *et* al. (2013) [50] | cohort | Y | Y | Y | Y | Y | Y | Y | Y | Y | Y | N | 22/22 |
| McGrail & Humphreys. (2015) [36] | cohort | Y | Y | Y | Y | Y | Y | Y | Y | N | U | Y | 19/22 |
| McGrail *et* al. (2016) [58] | cohort | Y | Y | Y | Y | Y | Y | Y | Y | N | U | Y | 19/22 |
| Murray *et* al. (2011) [28] | cohort | Y | Y | Y | N | N/A | Y | U | U | Y | Y | N | 14/22 |
| Nilsen *et* al. (2012) [29] | cohort | Y | Y | Y | N | N/A | Y | Y | Y | Y | Y | N | 16/22 |
| Norbye & Skaalvik. (2013) [30] | cohort | N/A | N/A | Y | N | N/A | Y | U | U | N | N | N | 6/22 |
| Opoku *et* al. (2015) [51] | cohort | Y | Y | Y | Y | Y | Y | Y | Y | Y | Y | Y | 22/22 |
| Patterson *et* al. (2016) [59] | cohort | N/A | N/A | Y | Y | N | Y | Y | Y | N | U | N | 11/22 |
| Pepper *et* al. (2010) [61] | cross-sectional | Y | N | Y | N/A | Y | Y | Y | Y | - | - | - | 12/16 |
| Playford *et* al. (2019) [60] | cohort | Y | Y | Y | Y | Y | Y | Y | Y | N | N | Y | 18/22 |
| Rabinowitz *et* al. (2013) [43] | cohort | Y | Y | Y | N | N/A | Y | Y | Y | Y | Y | N | 16/22 |
| Renner  *et* al. (2010) [33] | cohort | Y | Y | Y | Y | N | Y | Y | U | N | Y | N | 15/22 |
| Robinson & Slaney. (2013) [44] | cohort | N/A | N/A | Y | Y | N | Y | U | Y | Y | Y | N | 13/22 |
| Rodney *et* al. (2010) [45] | cohort | N/A | N/A | Y | Y | N | Y | Y | Y | Y | Y | N | 14/22 |
| Ross. (2013) [46] | cohort | N/A | N/A | Y | N | N/A | Y | Y | U | N | Y | N | 9/22 |
| Russell *et* al. (2013) [37] | cohort | Y | Y | U | Y | Y | Y | Y | Y | Y | Y | U | 20/22 |
| Straume *et* al. (2010) [52] | cohort | N/A | N/A | Y | N | N/A | Y | Y | Y | Y | Y | N | 12/22 |
| Wearne *et* al. (2010) [47] | cohort | N/A | N/A | Y | N | N/A | Y | U | Y | Y | Y | N | 11/22 |
| Woolley *et* al. (2017) [54] | cohort | Y | Y | Y | Y | Y | Y | Y | Y | N | Y | Y | 20/22 |
| Yong *et* al. (2018) [35] | cohort | Y | Y | Y | Y | Y | Y | Y | Y | N | Y | Y | 20/22 |
| Zhou. (2018) [34] | cohort and simulation | Y | Y | Y | Y | Y | Y | Y | Y | N/A | N/A | Y | 18/22 |

Responses to each question were assessed as “yes”, “no”, “unclear” or “not applicable” and scored as 2, 0, 1 and 0, respectively.

Y=yes; N=no; U=unclear; N/A=not applicable.

**JBI critical appraisal checklist for analytical cross-sectional studies**: Q1: Were the criteria for inclusion in the sample clearly defined? Q2: Were the study subjects and the setting described in detail? Q3: Was the exposure measured in a valid and reliable way? Q4: Were objective, standard criteria used for measurement of the condition? Q5: Were confounding factors identified? Q6: Were strategies to deal with confounding factors stated? Q7: Were the outcomes measured in a valid and reliable way? Q8: Was appropriate statistical analysis used?

**JBI critical appraisal checklist for cohort studies**: Q1: Were the two groups similar and recruited from the same population? Q2: Were the exposures measured similarly to assign people to both exposed and unexposed groups? Q3: Was the exposure measured in a valid and reliable way? Q4: Were confounding factors identified? Q5: Were strategies to deal with confounding factors stated? Q6: Were the groups/participants free of the outcome at the start of the study (or at the moment of exposure)? Q7: Were the outcomes measured in a valid and reliable way? Q8: Was the follow up time reported and sufficient to be long enough for outcomes to occur? Q9: Was follow up complete, and if not, were the reasons to loss to follow up described and explored? Q10: Were strategies to address incomplete follow up utilized? Q11: Was appropriate statistical analysis used?

**JBI critical appraisal checklist for case reports** (with questions adjusted to reflect a case as a health service rather than a patient)**:** Q1: Were health service characteristics clearly described? Q2: Was the health service history (including staffing) clearly described and presented as a timeline? Q3: Was the current health service retention clearly described? Q4: Were retention assessment methods and the retention outcomes clearly described? Q5: Was the retention intervention clearly described? Q6: Was the post-intervention retention outcome clearly described? Q7: Were unanticipated events identified and described? Q8: Does the case report provide takeaway lessons?
